# Effect of Vitamin D analog supplementation on levator ani strength and plasma Vitamin D receptor expression in uterine prolapse patients

**DOI:** 10.1038/s41598-023-30842-2

**Published:** 2023-03-03

**Authors:** Andi Kurniadi, Asri Kurnia Dewi, R. M. Sonny Sasotya, Benny Hasan Purwara, Jessica Kireina

**Affiliations:** Department of Obstetrics and Gynecology, Faculty of Medicine, Universitas Padjadjaran – Dr. Hasan Sadikin Hospital, Jl. Pasteur 38, Bandung, 40161 West Java Indonesia

**Keywords:** Urogenital diseases, Physiology, Anatomy

## Abstract

Uterine prolapse is a pathological condition that can negatively impact women's quality of life. It is caused by weakening of the pelvic floor muscles. Function of levator ani muscle and other striated muscles is thought to be influenced by Vitamin D levels. Vitamin D exerts its biological effects by adhering to Vitamin D receptors (VDRs) present in striated muscles. We aim to analyze the effect of Vitamin D analog supplementation on levator ani muscle strength in uterine prolapse patients. This was a quasi-experimental study with a pre-post design on 24 postmenopausal women diagnosed with grade III and IV uterine prolapse. Vitamin D levels, VDR, levator ani muscle, and hand grip muscle strength were measured before and after three months of Vitamin D analog supplementation. We found that Vitamin D level, VDR serum level, levator ani muscle strength, and hand grip muscle strength all significantly increase (*p* < 0.001) following Vitamin D analog supplementation. The correlation coefficient between levator ani muscle and handgrip muscle strength was 0.616 and with *p* value of 0.001. To conclude, Vitamin D analog supplementation can significantly increase levator ani muscle strength in uterine prolapse patients. We propose that determining Vitamin D level in postmenopausal women and replenishing its deficiency with Vitamin D analog supplementation might aid in preventing POP progression.

## Introduction

Uterine prolapse is one of multiple conditions that fall under the umbrella term of pelvic organ prolapse. It is characterized by descent of the uterus into the vagina due to weakness of endopelvic ligaments, particularly transverse ligaments. Pelvic organ prolapse can present as either vaginal prolapse, uterine prolapse, anterior wall prolapse or cystocele, and posterior wall prolapse or rectocele^1–4^.

The reported prevalence of POP varies greatly between studies and is estimated to be anywhere around 3–50%. These substantial differences are attributable to variations in study design, inclusion criteria, and accompanying symptom indicators^5^. The global prevalence of POP has been reported to be around 9%^6^. However in low-middle-income countries, this figure is closer to around 15%^7^.

Risk factors of POP include parity, method of delivery, previous pelvic surgery, BMI, conditions that increase intraabdominal pressure (chronic cough, constipation, heavy manual labor), age, and menopausal status. Prevalence of POP nearly doubles in postmenopausal and older women, with prevalence reaching 36–49% after 60 years of age^1,8^. While menopause is associated with collagen defects, aging affects POP by causing neuromuscular function and connective tissue changes. Aging is also correlated with gradual weakening of pelvic floor muscle, levator ani, and coccyx striated muscles. It is widely known that anatomical integrity and function of levator ani muscle play a significant role in pelvic organ support^9,10^. Women with levator ani disorders were found to be at least twice as likely to develop clinically significant POP and to experience recurrence after pelvic surgery^11,12^.

Unquestionably, the occurrence of POP can affect quality of life (QoL) and harms one’s physical, psychological, and social well-being. Pelvic organ prolapse is a particularly complex disorder that involves both physical and functional aspects. Women with POP may experience a range of urinary, gastrointestinal, and sexual symptoms that can seriously impact their QoL and mental health. Overall, women with prolapse had significantly lower QoL than the age-standardized population. The increase in life expectancy and the widespread movement towards improving quality of life have drastically increased the number of women seeking treatment and solutions to their symptoms^4,13–16^.Therefore, an effort to increase quality of life and prevent POP progression is continually being searched.

Vitamin D is a fat-soluble secosteroid hormone that regulates calcium, magnesium, and phosphate homeostasis; plays a pivotal role in bone growth; and acts as an antiproliferative and immunomodulatory mediator. In addition, Vitamin D has also been proven to affect muscle strength. Vitamin D exerts its biological effects by adhering to Vitamin D receptors (VDRs) that are also present in striated muscles like levator ani muscle. Through them, Vitamin D can affect striated muscle functions by regulating calcium homeostasis which would affect muscle contractility and help protect muscle cell environment from inflammation^1,2,17^.

The prevalence of Vitamin D deficiency among postmenopausal women worldwide ranges between 1.6 and 86%. Vitamin D deficiency can be a precipitating factor for pelvic floor damage in postmenopausal women^18–20^. An increase in Vitamin D is predicted to be able to help prevent pelvic organ prolapse progression. This study aims to analyze the effect of Vitamin D analog supplementation on levator ani muscle strength and plasma VDR expression in uterine prolapse patients.

## Materials and methods

This was a quasi-experimental study with a pre-post design that analyzes the effect of Vitamin D analog supplementation in women with uterine prolapse. We included all postmenopausal women diagnosed with grade III and IV uterine prolapse who came to outpatient clinic of Dr. Hasan Sadikin Bandung from August 2021 to November 2021. Uterine prolapse diagnosis and staging were based on Pelvic Organ Prolapse Quantification (POP-Q) system. Exclusion criteria were as follows:

- Patients with comorbidities, such as chronic cough and chronic constipation (these symptoms last for a minimum of 8 weeks).
- Patients diagnosed with diseases related to Vitamin D metabolism disorders such as: diabetes mellitus, chronic kidney failure, or malignant diseases
- Those who had a history of gastrectomy or jejunoileostomy surgery.
- Patients who are currently or have a history of taking cholesterol-lowering drugs (statins and fibrates), anticonvulsants, thiazides, theophylline, orlistat, cimetidine, and Vitamin D supplementation one month prior to this study.
- Patients withdrawing from our research.

During the initial presentation, we collected the following data: age, parity, body mass index (BMI), hemoglobin levels, and calcium levels. All subjects were then given 0.5 mcg of Vitamin D analog supplementation for 3 months. We also collected and compared the following data before and after Vitamin D analog supplementation: (1) Vitamin D and VDR serum levels, (2) Levator ani muscle strength, and (3) Hand grip muscle strength. Levator ani muscle strength was measured using perineometer, while handgrip muscle strength was evaluated using hand grip dynamometer.

We tabulated all patients' data on a customized spreadsheet and performed data analysis on Statistical Produce and Service Solutions SPSS software version 25 for Windows (IBM Corp, Armonk, New York, USA). Descriptive statistics were performed as appropriate. Analytical statistics were performed using t test or Wilcoxon test as required, with *p* < 0.05 considered as significant.

Written informed consent was provided to all study participants prior to engaging in any study-related procedures. Ethical approval of this study was granted by the Health Research Ethics Committee of Hasan Sadikin Hospital, Bandung under the following registration number: LB.02.01/X.6.5/213/2021. This study was conducted according to Declaration of Helsinki. All research procedures were performed in accordance with relevant guidelines and regulations.

## Results

We recruited 24 subjects. Most of them were in the age group of 50–70 years old, had parity ≥ 3, and had normal BMI. Mean hemoglobin was 12.82 ± 1.75 and mean calcium level was 4.87 ± 0.42 (Table 1).

**Table 1:** Characteristics of subjects.

Variables	Subjects (n = 24)
Age	
< 50 years	2 (8.3%)
50–70 years	18 (75.0%)
> 70 years	4 (16.7%)
Parity	
< 3	6 (25.0%)
≥ 3	18 (75.0%)
Body mass index (BMI)	
Normal	17 (70.8%)
Overweight	6 (25.0%)
Obesity	1 (4.2%)
Hemoglobin	
Mean ± SD	12.82 ± 1.75
Median (range)	13.10 (8.50–16.80)
Calcium	
Mean ± SD	4.87 ± 0.42
Median (range)	4.89 (4.05–5.56)

We found that levator ani muscle strength significantly increases following Vitamin D analog supplementation (10,00 ± 3,64 to 19,68 ± 3,70, *p* = 0.001). A similar significant result was also found when testing for handgrip muscle strength after Vitamin D analog supplementation (15,39 ± 3,36 to 23,87 ± 3,81, *p* = 0.001). Both Vitamin D and VDR serum levels also significantly increase following Vitamin D analog supplementation (both *p* = 0.001, Table 2). In addition, we also assessed the correlation between levator ani muscle and handgrip muscle strength and found a significant positive correlation (r = 0.616, *p* = 0.001), indicating that the stronger the levator ani muscle, the stronger the handgrip muscle.

**Table 2:** Comparison of levator ani muscle strength, hand grip muscle strength, Vitamin D, and VDR before and after Vitamin D supplementation.

Variable	Before supplementation	After supplementation	*p* value
Levator ani muscle strength	10.00 ± 3.64	19.68 ± 3.70	0.001*
Hand grip muscle strength	15.39 ± 3.36	23.87 ± 3.81	0.001*
Vitamin D	8.90 (6.20–16.80)	10.90 (7.40–26.50)	0.001**
VDR	0.05 (0.01–0.17)	0.10 (0.05–1.10)	0.001**

*Dependent t test.

**Wilcoxon test.

## Discussion

Uterine prolapse is a pathological condition caused by weakening of the pelvic floor muscles. Decreased neuromuscular function and connective tissue changes in older and postmenopausal women are associated with decreased muscle function and therefore uterine prolapse incidence^1–4^. There were 24 subjects in this study and most of them were in the age group of 50–70 years old, had parity ≥ 3, and had normal BMI. Age, parity, and BMI are widely known risk factors of POP^2–4,12^.

Aging is the most widely reported contributing factor to pelvic organ prolapse^14^. As age increases, striated muscle strength becomes weaker and muscle regeneration process grows slower. Furthermore, as people age, their muscle mobility and pelvic supporting tissue integrity decrease^8,21,22^. 

Seven out of 24 subjects had BMI above normal. Excess body mass index is considered a risk factor for uterine prolapse because it correlates with levator ani muscle weakness^21,23^. A study by Maclennan et al. found that obesity was significantly associated with pelvic floor muscle strength^21^. Obese women (BMI > 30) have a two-fold risk of experiencing POP compared to those with normal BMI^24^.

Most of the subjects were multiparous, with parity of ≥ 3. In 2013, Yeniel et al. conducted a study and found that multiparous women had 2.92 times greater risk of developing POP than nulliparous women. This same study also found that the risk of POP in each subsequent delivery increased by 1.2 times^25^. In addition, another study in 2017 stated that vaginal delivery was associated with worse pelvic organ support five years following first delivery^26^.

All subjects in this study had Vitamin D deficiency since the beginning of the study. Our findings are in line with previous studies on Vitamin D levels in postmenopausal women. According to a study conducted in China, the incidence of Vitamin D deficiency among postmenopausal women is approximately 99.4 percent. A study conducted in Bandung and Sumedang, Indonesia, revealed that 94.3% of postmenopausal women lacked Vitamin D^19,20,27^.

A vast body of evidence suggests that abnormalities of Vitamin D level and its signaling may play a significant role in the development of obstetric and gynecological disorders in various age periods of a woman’s life. Vitamin D level abnormalities have been linked to diseases like PCOS, endometriosis, ovarian cancer, breast cancer, infertility, and pelvic organ prolapse, especially in postmenopausal women^28^. The link between Vitamin D abnormalities and pelvic organ prolapse development lies in Vitamin D’s function on striated muscles. Previous studies have extensively documented the effects of Vitamin D on the strength and function of striated muscle. Improved physical performance has been linked to Vitamin D. Conversely, muscle weakness and sarcopenia are associated with Vitamin D deficiency. Pelvic floor muscle weakness can manifest clinically as pelvic floor dysfunction symptoms. Levator ani and coccygeus muscles are important components of the pelvic floor and can be affected by Vitamin D status^19,20,29,30^. Our results indicated that Vitamin D deficiency could be one precipitating factor for pelvic floor damage in postmenopausal women^20–22^. This is supported by previous studies in America, Canada, and Europe which showed that Vitamin D deficiency is an important factor in POP occurrence^31^.

In this study, levator ani muscle contraction strength before Vitamin D analog supplementation was 10.00 ± 3.64 cmH2O with a range of 4.70–17.00. After Vitamin D analog supplementation, levator ani muscle contraction strength increased significantly to 19.68 ± 3.70 cmH2O with a range of 10.70–25.60 (*p* = 0.001). Pelvic organ prolapse can be treated both medically and surgically. Pessaries, pelvic floor muscle training, or both can be used as non-invasive treatments to help alleviate POP symptoms. The goal of pelvic muscle training is to strengthen pelvic floor muscle (including levator ani) and help improve POP symptoms, particularly urinary incontinence. One previous study mentioned that pelvic floor muscle training guided by a motion-based digital therapy device can significantly improve urinary incontinence and decrease its episodes^32^. The result of our study suggested that Vitamin D analog supplementation can strengthen levator ani muscle contraction and thus aid in improving POP symptoms and preventing its progression.

We also found a significant increase in hand grip muscle strength following three months of Vitamin D analog supplementation (*p* = 0.001). Prior to supplementation, mean hand grip muscle strength was 15.39 ± 3.36 (range = 10.00–21.00), then after supplementation, hand grip muscle strength increased to 23.87 ± 3.81 (range = 16.70–30.40). A study conducted by Setiati et al., reported a similar result to our study, where alfacalcidol (Vitamin D analog) was found to increase hand grip muscle strength in elderly. The hand grip strength correlates with total muscle strength and can be used to assess an individual's overall muscle strength^30^. Furthermore, we also found a positive correlation between levator ani muscle and handgrip muscle strength meaning that the stronger the levator ani muscle, the stronger the handgrip muscle.

Ceglia et al. reported that Vitamin D may affect striated muscle through both genomic and nongenomic mechanisms. In the genomic pathway, Vitamin D regulates gene transcription by binding to Vitamin D receptors on the nuclear membrane of muscle cells, resulting in the differentiation and proliferation of muscle cells through the effects of insulin growth factor (IGF), which eventually induces muscle hypertrophy. Whereas in the nongenomic pathway, Vitamin D (1,25(OH)D) binds to receptor membrane which then activates signal transduction, triggering the MAP Kinase (MAPK) and Phospholipase C (PLC) pathways, and causing a rapid influx of calcium into cells and affecting muscle cell contraction^33^.

In conclusion, Vitamin D analog supplementation can significantly increase levator ani muscle strength. We propose that determining Vitamin D level in postmenopausal women and replenishing its deficiency with Vitamin D analog supplementation might help strengthen pelvic floor muscle and therefore aid in preventing POP progression.

This is a preliminary study that analyzes the effect of Vitamin D supplementation towards pelvic floor muscle strength, particularly levator ani muscle strength. Further clinical studies on the effect of Vitamin D supplementation towards rate of recurrence and risk of surgery are needed. In addition, future studies should also look at the dosage of Vitamin D supplementation needed based on prolapse severity.

## Data Availability

The datasets used and/or analyzed during the current study are available from the corresponding author upon reasonable request.
